# Botch Bosses

**Published:** 1890-06

**Authors:** S. H. Preston


### BOTCH BOSSES.

BY S. H. PRESTON.

The great need of the day is mechanics who are masters of their calling. Among American trades there is a lamentable lack of first class workmen. The trouble is, people do not find time to thoroughly learn a trade. A boy is "bound out," before he hardly gets a smattering of his business, off he goes and sets up shop for himself. He determines to be independent, to boss his own business. And now he must procure patronage or starve. So he must needs buy a poor set of tools and cheap stock of material, and "cut under" the customary price, so as to secure work. The community soon find out that he is a botch and let him alone. Failing to get any thing to do, he learns to lounge around lager beer saloons, and is finally forced to shut up shop and become a tramp.

The majority of American mechanics are poor, being scarcely able to maintain their families. This is mainly because so many are incompetent. They not only doom themselves to lack of work and low wages, but bring disrepute upon their craft. There are plenty of people who are deterred from making improvements and repairs because they find it so difficult to get a good job done.

Now, who ever knew of a *good* workman in any branch of business, who did not have all he could do? Go to a *good* mechanic and you generally have to "wait your turn." They are not only always in demand, but can command good prices, make money, and are usually "forehanded." They dignify their craft, and elevate themselves and families. Were there more of them, more work and better wages would be the result.

Well, what is the remedy? Simply the enforcement of a rigid system of apprenticeship, something like that prevailing in European countries to-day, or which prevailed in this country in the days of our fathers. In other countries, a mechanic must pass from three to seven years in mastering his trade.

Skilled labor is worth as much here as elsewhere. But under our present lax system of apprenticeship, the number of expert, competent mechanics is decreasing. We need a law prescribing a certain number of years to be served at such and such trades, and prohibiting a person pursuing an occupation for himself, till he is the perfect master of it.

And every father who intends his son to follow a trade, should be made to see that the son serves his full term of apprenticeship, or else be compelled to provide for him till competent in his calling. This will preclude botches, increase work, raise wages, dignify labor, inspire confidence in employers, and promote enterprise, thrift and industry.

